# Modulation of social behavior by the agouti pigmentation gene

**DOI:** 10.3389/fnbeh.2014.00259

**Published:** 2014-08-01

**Authors:** Valeria Carola, Emerald Perlas, Francesca Zonfrillo, Helena A. Soini, Milos V. Novotny, Cornelius T. Gross

**Affiliations:** ^1^IRCCS Fondazione Santa LuciaRome, Italy; ^2^Mouse Biology Unit, European Molecular Biology LaboratoryMonterotondo, Italy; ^3^Department of Chemistry, Institute for Pheromone Research, Indiana UniversityBloomington, IN, USA

**Keywords:** social behavior, agouti, melanocortin receptors, preputial glands, volatile organic compounds (VOCs), sorptive stir bar extraction (SBSE)

## Abstract

Agouti is a secreted neuropeptide that acts as an endogenous antagonist of melanocortin receptors. Mice and rats lacking agouti (called non-agouti) have dark fur due to a disinhibition of melanocortin signaling and pigment deposition in the hair follicle. Non-agouti animals have also been reported to exhibit altered behavior, despite no evidence for the expression of agouti outside the skin. Here we confirm that non-agouti mice show altered social behavior and uncover expression of agouti in the preputial gland, a sebaceous organ in the urinary tract that secretes molecules involved in social behavior. Non-agouti mice had enlarged preputial glands and altered levels of putative preputial pheromones and surgical removal of the gland reversed the behavioral phenotype. These findings demonstrate the existence of an autologous, out-of-skin pathway for the modulation of social behavior.

## Introduction

The agouti gene expresses a secreted neuropeptide that acts as an endogenous antagonist of melanocortin receptors (Lu et al., 1994; Logan et al., 2003) and is involved in the suppression of melanin deposition during the hair cycle to produce the characteristic striped brown color of mouse and rat fur (Bultman et al., 1992; Millar et al., 1995). Mutations that result in the ubiquitous overexpression of agouti lead to the widespread blockade of melanocortin receptor signaling (Furumura et al., 1996) demonstrating that agouti is a non-specific antagonist of melanocortin receptors. Behavioral geneticists have successfully used correlations between behavioral traits and external morphometric features to classify and identify genetically controlled traits and investigate pleiotropic phenotypes (Arcus and Kagan, 1995; Hayssen, 1997). Several investigators have noted that coat color in rodents is associated with differences in behavior, and studies on wild mice and rat strains reported differences in both escape and aggressive behavior between non-agouti and agouti animals (Cottle and Price, 1987; Hayssen, 1997).

During an assessment of behavioral differences between mice of the C57BL/6J and BALB/cByJ inbred strains we noticed a significant difference in locomotor behavior (Carola et al., 2006) and found that this phenotype segregated with the agouti locus in F2 intercrosses between these strains (data not shown). The C57BL/6J strain harbors a null allele of agouti (*a*, non-agouti) and the BALB/cJ strain carries the putative wild-type agouti allele (*A*, white-bellied agouti). To confirm if the non-agouti allele was responsible for behavioral differences between these strains, we examined the behavior of a spontaneous revertant strain of non-agouti, called *A*^w−J^ (white-bellied agouti, JAX), in which the viral element that blocks expression of the gene in the non-agouti allele (Bultman et al., 1994) has been excised. In light of the evidence that agouti expression antagonizes the action of α-melanocortin stimulating hormone (α-MSH), a hormone that modulates aggression behavior in mice (Nowell and Wouters, 1975; Nowell et al., 1980; Morgan and Cone, 2006), we decided to test social behavior in this spontaneous revertant strain. Our findings demonstrate that null mutations in the agouti gene are associated with increased sociability and aggression in mice, despite the absence of expression of this gene in the central nervous system. Unexpectedly, we found agouti mRNA expression in the preputial gland and surgical removal of this organ reversed the mutant phenotype in non-agouti mice. These findings uncover a novel, out-of-skin mechanism by which an organism can regulate its own social behavior.

## Materials and methods

### Animals

Male and female C57BL/6J-*A*^w−J^/J (*A*, agouti) and C57BL/6J (*a*, non-agouti) mice were obtained from The Jackson Laboratory (Bar Harbor, ME). Breeding to obtain agouti and non-agouti littermates for behavioral and biochemical phenotyping was performed between heterozygous agouti male and homozygous non-agouti female mice at 8–10 weeks of age. Male CD1 mice were obtained from Charles River (Calco, Milan, Italy) and used at 12 weeks of age. Mice were housed in groups of 2–3/cage (35 × 15 × 12 cm) at constant temperature (21 ± 1°C) and humidity (55 ± 5%). Food and water were provided *ad-libitum*. Mice were housed on a 12:12 light/dark cycle (lights on at 7:00) until 3 weeks before behavioral testing, when the mice were moved to a room close to the testing room and switched to a reverse 12:12 dark/light cycle (lights off at 10:30) for the duration of the testing period. All behavioral testing was performed during the dark cycle from 15:30 to 17:30 under dim red light illumination (two 60 W lamps). Different groups of mice were tested in each behavioral test. All work with animals was performed in accordance with European guidelines for the ethical treatment of animals and under approval of the Italian Ministry of Health (#25/2004-B, #91/2007-B, #231/2011-B).

### Behavioral testing

#### Resident-intruder test

Agouti and non-agouti male mice were single housed for 3 weeks before being tested in the resident-intruder paradigm at 12–14 weeks of age. Intruders were grouped-housed non-agouti male mice. Cages of resident and intruder mice were moved to the testing room 40 min before the test. At the beginning of the test, an intruder mouse was placed into a resident cage and the behavior of both mice observed for 40 min. The test was repeated once a week for 3 consecutive weeks. Different intruders were used each time to avoid habituation. On the fourth week an extra trial was carried out where half of each group of resident mice was exposed to an agouti and the other half to a non-agouti intruder mouse. *Latency to the first attack* and *number of attacks* of the resident mouse were collected in real-time during the test. A similar resident-intruder test was performed on a separate group of mice with CD1 outbred intruders (see Supplementary Material).

#### Social interaction test

In order to habituate animals to the testing apparatus and avoid confounds associated with social hierarchies arising within group housed mice, one agouti and one non-agouti male mouse were housed together in a single cage, separated by a perforated plexiglass barrier for a week before being tested in the social interaction test at 12–14 weeks of age. At the beginning of the test, the barrier was removed and the behaviors of the two mice were scored once every minute for 20 min. Testing was performed daily for 4 consecutive days. Behavioral parameters of the two mice were manually scored during each session, and included: *digging,rearing*, *jumping, sniffing environment* (olfactory exploration of the cage), *social sniffing* (olfactory exploration of the other mouse), *social grooming* (licking/scratching fur of the other mouse), *self-grooming, pursuit* (close following of the other mouse), *escape*, *defensive posture,* and *attack.*

#### Olfactory approach test

Grouped housed agouti and non-agouti mice were moved to the testing room 30 min before the test. During the training session (15 min) each mouse was free to explore the apparatus, a gray plexiglass box (30 × 20 × 20 cm) with two compartments connected by a small opening into which two plastic petri dishes (35 mm diameter) had been placed. At the end of the training each mouse was confined to one of the two compartments by a removable divider and the dish in the other compartment was filled with water. The procedure was repeated for the other compartment but this time the dish was filled with 200 μl of adult male mouse urine. This side of the apparatus was defined as “social compartment/side.” During the testing phase the mouse was left free to explore the two compartments for 15 min. This experiment was repeated twice, once using urine from non-agouti mice and once using urine from agouti mice. Locomotion data were collected by a video-tracking system (TSE Systems, Bad Homburg, Germany). Social preference was calculated by subtracting the time spent in the same compartment during the training phase (in the absence of urine) to the time spent in the social compartment during the testing phase (in the presence of urine). Urine marks were collected during the “testing” phase of the olfactory approach test. Two pieces of filter paper were placed on the floor the apparatus (one in each compartment) and urine marks were viewed under ultraviolet light as previously described (Desjardins et al., 1973). We counted the total number of the separate urine marks deposited and the area covered with urine was estimated by placing a transparent grid sheet over the filtered paper and counting the total number of grids (1 cm^2^) containing the scent marks.

#### Open field test

Mice were habituated to the room for 40 min before being placed into a gray plastic arena (50 × 50 cm) for 40 min. Locomotion data were collected by a video tracking system (TSE). Animals were initially placed along one side of the arena.

#### Elevated plus maze test

Mice were habituated to the room for 10 min before being placed into the central platform (5 × 5 cm) of a gray plastic maze facing toward an open arm (open arm: 30 × 5 cm, surrounded by a 0.25-cm high border, closed arms: 30 × 5 cm, surrounded by 15-cm high walls). The entire apparatus was elevated 45 cm above the floor. Locomotion data were collected for 5 min by a video TSE.

### Preputial gland weight analysis

Preputial glands were collected and weighed from a total of 42 mice: 25 single and 17 group-housed. In the single-housed mice the collection was performed at the end of a 2-weeks isolation period.

### Surgery

Surgical removal of preputial glands from group-housed mice was carried out according to published protocols (Morgan and Cone, 2006). After surgery, mice were singly housed and left to recover for 3 weeks. After recovery mice were tested a second time in the social interaction test.

### *In situ* hybridization

Preputial glands and skin from adult agouti and non-agouti mice were dissected, fixed overnight in 4% paraformaldehyde, and embedded in paraffin. *In situ* hybridization using digoxigenin-labeled or ^35^S-CTP-labeled probes on 8 μm paraffin sections was performed according to procedures previously described (Neubüser et al., 1995; Niederreither and Dollé, 1998). Briefly, sections were de-waxed, rehydrated, digested with proteinase K, and hybridized with probe at 65°C. Posthybridization washes in 20% formamide, 0.5 SSC were done at 60°C. The mouse *agouti* cDNA clone was a gift of Gregory Barsh (Department of Genetics, Stanford University, Palo Alto, CA). Adjacent 8 μm thick sections were used for Pyronin Y staining. The sections were deparaffinized, and rehydrated to distilled water before 5 min staining in 0.05% pyronin Y in 0.2 M acetate buffer.

### Analysis of volatile organic compounds (VOCs)

#### Standard materials

When available, compound identifications were verified through authentic standards purchased from Aldrich Chemical Company (Milwaukee, WI, USA) and Alfa Aesar (Johnson Matthey Inc., Taylor, MI).

#### Sample preparation

The preputial glands surgically removed at the end of the olfactory approach test (**Figure 2H**) were used for the volatile organic compound (VOC) analysis. Each gland was weighed, frozen by adding a small amount of liquid nitrogen followed by homogenization with mortar and pestle. The powdered gland was then dispersed into 20 mL of the 5% ethanol-water solution and placed in the 20-mL scintillation vial. For the stir bar sorptive extraction (SBSE) of VOCs (Soini et al., 2005), a Twister® stir bar (10 mm in length, 0.5 mm film thickness, 24-μL polydimethylsiloxane, PDMS, volume) was added into the vial (Gerstel GmbH & CoKG, Mühlheim an der Ruhr, Germany). The used stir bars were conditioned prior to and between individual runs in the TC-2 tube conditioner (Gerstel) at 300°C under the high flow of purified helium. After the 60-min extraction (800 rpm, Variomag stir plate, Variomag-USA, Daytona, FL), the stir bar was rinsed with ultrapure OmniSolv® water (EMD, Millipore, Billerica, MA), dried gently and placed in the thermal desorption tube in the TDSA autosampler (Gerstel). Samples were desorbed in the TDSA system, followed by injection into the column with a cooled injection assembly, CIS-4 (Gerstel). Temperature program for TDSA desorption was 20°C (0.5 min), then 60°C/min to 280°C (10 min). Temperature of the transfer line was set at 280°C. The CIS was cooled with liquid nitrogen to −80°C. After desorption and cryotrapping, the CIS was heated at 12°C/s to 280°C with the hold time of 10 min. The CIS inlet was operated in the solvent-vent mode, a vent pressure of 9 psi, a vent flow of 30 mL/min, and a purge flow of 50 mL/min.

#### Gas chromatography-mass spectrometry

The GC-MS instrument used for the compound identification and quantification was an Agilent 6890N gas chromatograph connected to an 5973i MSD mass spectrometer (Agilent Technologies, Wilmington, DE). The GC column was DB-5MS (30 m × 0.25 mm, i.d., 0.25 μm film thickness, Agilent Technologies). The inlet head pressure was 9 psi for the helium flow of 1.2 mL/min. The GC oven temperature program was 40°C for 5 min, followed by a ramp of 2°C /min to 200°C (hold time 10 min). The system operated in the constant-flow mode. Positive electron ionization (EI, 70 eV) mode was used with the scanning rate of 2.41 scans/s over the mass range of 41–350 amu. The MSD transfer line temperature was set at 280°C. The ion source and quadrupole temperatures were set at 230 and 150°C, respectively.

#### Quantitative comparisons

For quantitative comparisons, peak areas were normalized by dividing peak areas of each separated component by the peak area of the internal standard (7-tridecanone) in the same analytical run. GC-MS total ion currents (TICs) were used for these calculations. Additionally, the obtained values were divided by the gland weight (normalized by weight).

### Statistical analysis

The effects of genotype and day on behavioral variables were analyzed by repeated measure analysis of variance (ANOVA). Analysis of the effect of genotype on preputial gland secretion and behavior was performed by ANOVA and in cases of significance (*P* < 0.05) followed by *post-hoc* comparisons using Duncan's test. Analysis of the effect of genotype and housing condition on preputial gland weight was performed by ANOVA.

## Results

### Non-agouti mice show altered social behavior

**Figure 1:**
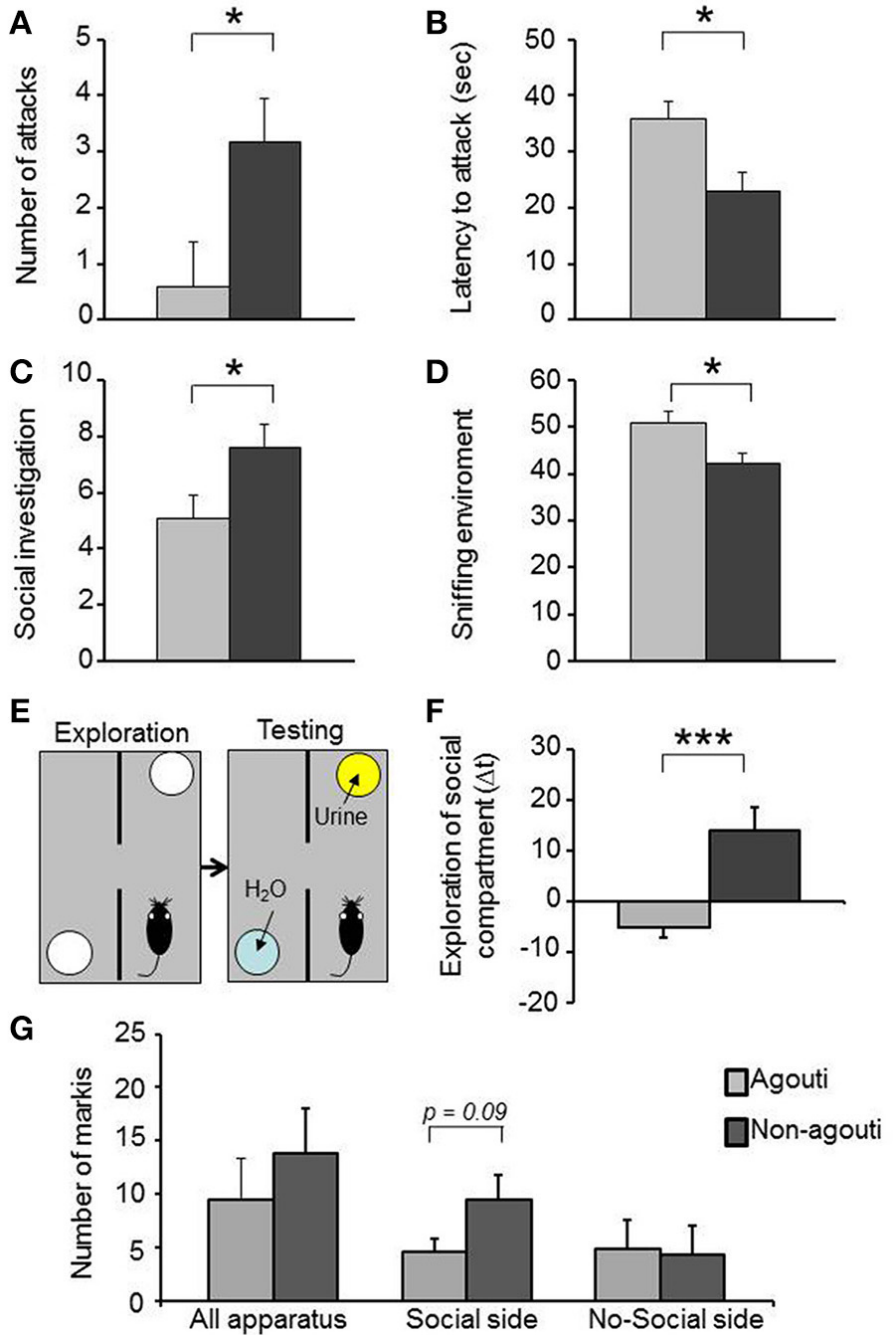
**Non-agouti mice are more sociable**. Male non-agouti mice performed significantly more **(A)** attacks and showed a significantly shorter **(B)** latency to the first attack than male agouti littermates in the resident-intruder test (*N* = 10). Male non-agouti mice performed significantly more **(C)** social exploration and significantly less **(D)** environmental exploration than male agouti littermates in the social interaction test (fraction of total observations; *N* = 14). **(E)** In the olfactory approach test mice were allowed to explore two compartments during an initial exploration phase followed by a testing phase in which urine from an adult male mouse was placed into one compartment. **(F)** Male non-agouti mice showed a significantly greater preference for the urine-containing compartment when compared to male agouti littermates. (*N* = 12–13, ^*^P < 0.05, ^***^P < 0.001). **(G)** Non-agouti mice tended to perform more scent marking than agouti mice in the urine-containing side of the apparatus used for the olfactory approach test.

Non-agouti mice showed decreased locomotion in the open field and elevated plus maze tests when compared to agouti littermates (Figure S1). We also examined aggressive-like behavior in the resident-intruder test. Non-agouti mice showed significantly increased aggressive-like behavior when compared to agouti littermates in the test, exhibiting more attacks [Figure 1A; repeated measure ANOVA, genotype effect: *F*_(1, 18)_ = 5.40, *P* = 0.032] and a shorter latency to the first attack [Figure 1B; repeated measure ANOVA, genotype effect: *F*_(1, 18)_ = 7.77; *P* = 0.012] toward a non-agouti intruder over three consecutive trials. To evaluate if aggressive behavior of the resident could be modulated by the genotype of the intruder a fourth trial was carried out in which each group was split and half were exposed to non-agouti and the other half to agouti intruders. No significant behavioral differences between mice exposed to agouti or non-agouti intruders were detected (Figure S2). Finally, we examined the possibility that the agouti genotype of a mouse might elicit different levels of aggression in an opponent by performing a resident-intruder test with CD1 outbred intruder mice, a strain characterized by high levels of aggressive behavior (see Supplementary Material). Under these circumstances CD1 intruders invariably attack the residents during the first encounter. However, no difference was observed in the number of attacks or latency to attack toward non-agouti and agouti residents suggesting that the indirect genetic effects of agouti on aggression are minimal (Figure S3).

Non-agouti mice also showed a significant increase in social investigation [Figure 1C; repeated measure ANOVA, genotype effect: *F*_(1, 26)_ = 5.18, *P* = 0.031] and decrease in exploration of the environment [Figure 1D; repeated measure ANOVA, genotype effect: *F*_(1, 26)_ = 7.69, *P* = 0.010] in a social interaction test where the mouse is confronted with a stranger mouse in a novel environment. To determine whether the increased social investigation was dependent on an intrinsic change in social behavior of the experimental animal or might have arisen from a change in behavior elicited in the opponent, we measured approach behavior toward a social olfactory cue. Non-agouti mice spent significantly more time in close proximity to a dish of urine from an unfamiliar male mouse than agouti littermates [Figures 1E,F; ANOVA, genotype effect: *F*_(1, 23)_ = 14.79, *P* = 0.001] and tended to perform more scent marking behavior in the urine-containing side of the apparatus [Figure 1G; ANOVA, genotype effect: *F*_(1, 11)_ = 3.46, *P* = 0.090]. The preference for the urine-containing compartment was independent of the type of urine used, with both genotypes showing similar behavior in the presence of non-agouti or agouti urine [Figure S4; ANOVA genotype effect: *F*_(1, 31)_ = 16.89, *P* = 0.001].

### Expression of agouti in preputial gland

**Figure 2:**
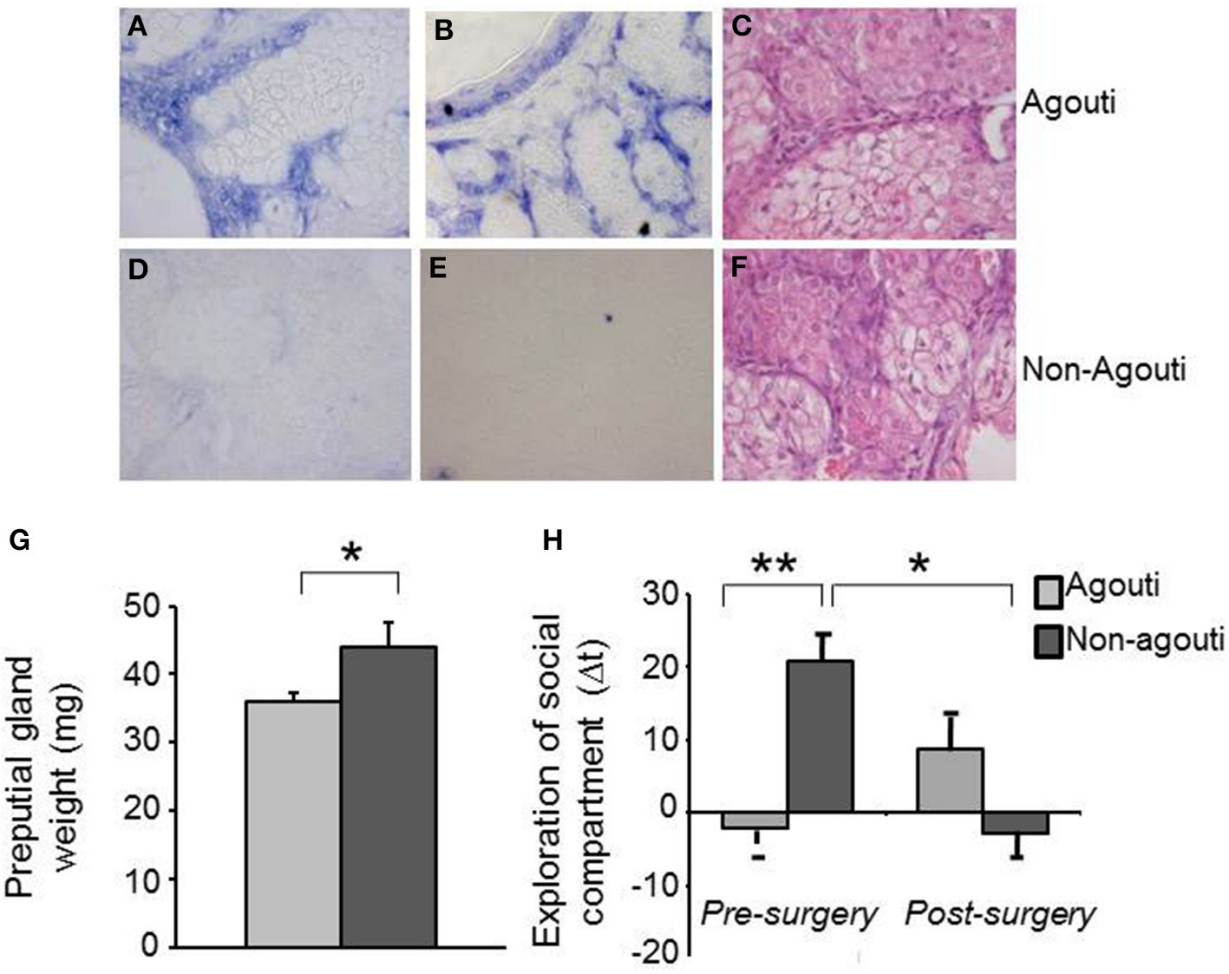
**Preputial gland is required for the behavioral effects of agouti**. Representative images of *in situ* hybridization data show specific expression of agouti mRNA in cells of preputial gland tissue from **(A,B)** agouti, but not **(D,E)** non-agouti mice. Representative images of pyronin Y staining show similar tissue structure in preputial gland of **(C)** agouti and **(F)** non-agouti mice. **(G)** Preputial gland weight was significantly increased in non-agouti mice when compared to agouti littermates (*N* = 21–22). **(H)** Non-agouti mice showed significantly higher preference for the compartment containing male urine in the olfactory approach test when compared to agouti littermates. Preputialectomy significantly reduced preference for the urine-containing compartment in non-agouti, but not agouti mice in this test (*N* = 12; ^*^P < 0.05, ^**^P < 0.01).

Agouti mRNA has not been reported in the central nervous system, we therefore explored the possibility that expression in the skin might be responsible for the effects of agouti on social behavior we had observed. We checked the agouti mRNA in cells of the preputial gland, a specialized sebaceous organ responsible for the secretion of molecules with pheromone properties into the urine, by *in situ* hybridization and interestingly we observed expression only in agouti but not non-agouti males (Figures 2A–F). This result demonstrates that agouti is expressed in skin appendages other than the hair follicle and suggest that it may have an effect on melanocortin receptor signaling in the preputial gland. Consistent with an antagonistic effect of locally produced and secreted agouti protein on Mcr5 activity, preputial glands from non-agouti mice were significantly heavier than those from agouti littermates [Figure 2G; ANOVA, genotype effect: *F*_(1, 41)_ = 4.50, *P* = 0.040]. An independent effect of the housing condition on the preputial gland weight was also observed [ANOVA, housing effect: *F*_(1, 41)_ = 5.29, *P* = 0.030] but no interaction between genotype and housing condition [ANOVA, genotype × housing condition effect: *F*_(1, 41)_ = 0.55, *P* = 0.460].

### Differences in preputial gland volatile organic compounds

**Figure 3:**
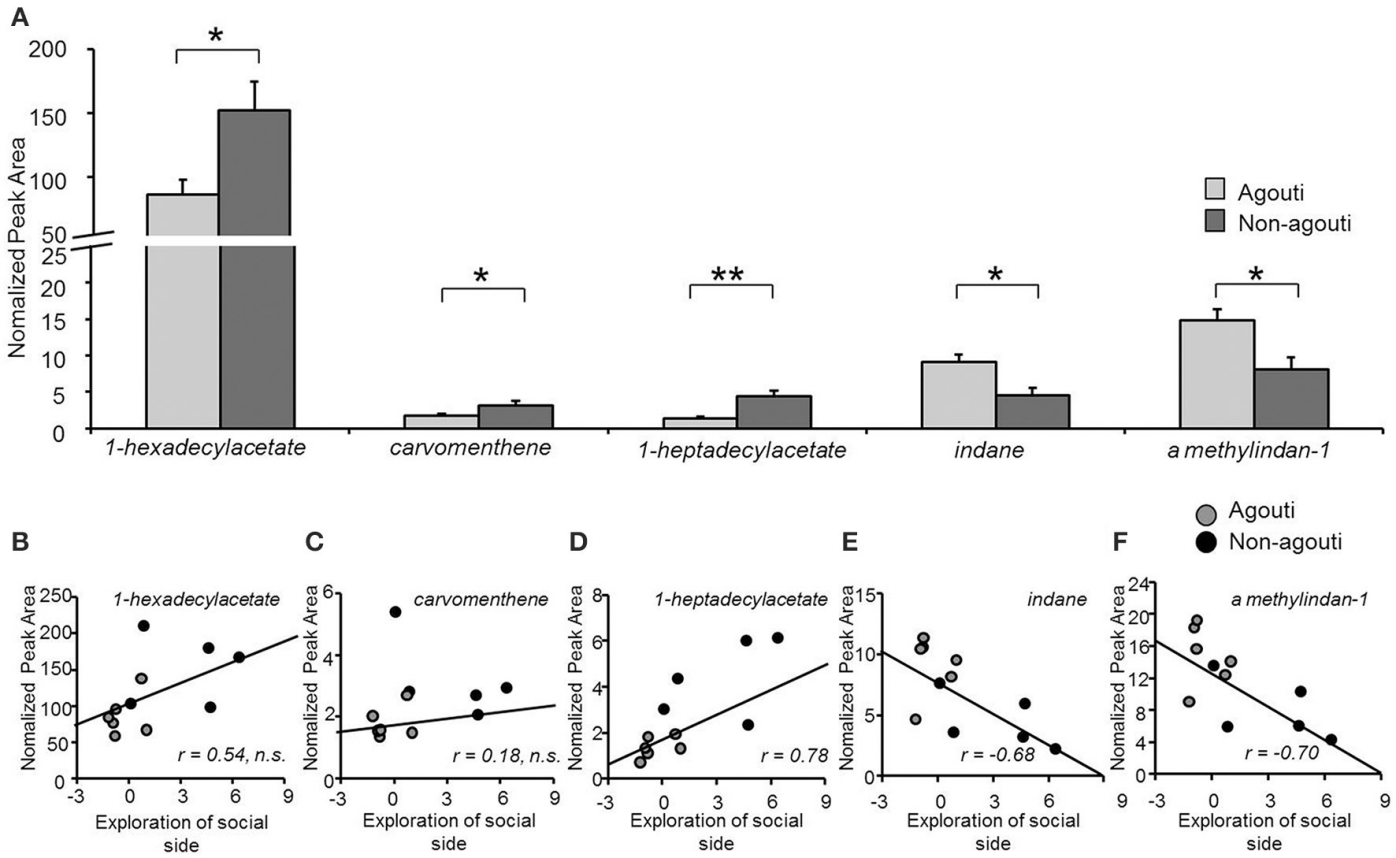
**Secretion from preputial gland is different between non-agouti and agouti mice. (A)** Non-agouti mice showed significant higher levels of 1-hexadecylacetate, carvomenthene, 1-heptadecylacetate and lower levels of indane and A methylindan-1 in the preputial glands compared to agouti mice. **(B–F)** Correlation analyses performed between the levels of these compounds (normalized peak area) and the preference for the urine-containing compartment (social side) measured in the same mice in the olfactory approach test, showed that the levels of heptadecylacetate, indane, and A methylindan-1 were significantly correlated with this behavior. (*N* = 11; ^*^P < 0.05, ^**^P < 0.01).

To examine whether agouti genotype might have an effect on social behavior by altering the production of pheromone-like substances in the preputial gland, we used SBSE/GC-mass spectrometry to quantify VOC from preputial glands collected from agouti and non-agouti mice following testing in the olfactory approach test. VOC profiles obtained by the method contained about 100 compounds of which 21 could be identified and their normalized levels compared quantitatively (Table S1). We observed that non-agouti mice had higher levels of 1-hexadecylacetate [ANOVA, genotype effect: *F*_(1, 10)_ = 7.58, *P* = 0.022], a compound found in higher quantities in males than females and reported to promote social investigation in a dose and sex-dependent manner (Zhang et al., 2007, 2008; Figure 3A). We also found higher levels of carvomenthene [ANOVA, genotype effect: *F*_(1, 10)_ = 6.25, *P* = 0.040] and 1-heptadecylacetate [ANOVA, genotype effect: *F*_(1, 10)_ = 17.22, *P* = 0.002] and lower levels of indane [ANOVA, genotype effect: *F*_(1, 10)_ = 10.55, *P* = 0.040] and A methylindan-1 [ANOVA, genotype effect: *F*_(1, 10)_ = 8.67, *P* = 0.016] in non-agouti compared to agouti mice (Figure 3A). Absolute levels of some (1-heptadecylacetate, indane, A methylindan-1), but not all of these compounds were significantly correlated with exploratory behavior in the olfactory approach test across animals (Figures 3B–G) consistent with a possible causal link between preputial gland volatile production and social behavior.

### Reversal of behavioral phenotype by preputialectomy

To test whether changes in preputial gland function might underlie the social behavior phenotype of non-agouti mice we examined social behavior before and after removal of the preputial gland in non-agouti and agouti mice. Surprisingly, preputialectomy did not significantly alter social approach behavior in agouti mice. However, preputialectomy of non-agouti mice caused a significant reduction of social approach behavior and reversed the behavioral phenotype seen in these mice [Figure 2H; repeated measure ANOVA, surgery × genotype effect: *F*_(1, 10)_ = 6.49, *P* = 0.026].

## Discussion

Our findings of altered locomotor and social behavior in non-agouti mice are consistent with a causal role for the agouti gene in the strain differences previously reported between agouti and non-agouti animals (Cottle and Price, 1987; Hayssen, 1997; Yamamuro and Shiraishi, 2011). Previous attempts to identify agouti mRNA expression in the central nervous system failed (Vrieling et al., 1994; Millar et al., 1995) leaving open the mechanism by which agouti might modulate behavior. Our finding that agouti is expressed in the preputial gland, a sebaceous-like secretory organ, is consistent with its well-documented expression in skin appendages and for the first time offers a mechanism for how agouti might regulate neuronal physiology.

Although our data demonstrate a critical role for the preputial gland in modulating the social behavior of its host, several features of this modulation remain to be worked out. Previous reports have shown that α-MSH-induced preputial hypertrophy is associated with the increased release of pheromones that promote aggression (Nowell and Wouters, 1975; van der Kraan et al., 1998; Morgan and Cone, 2006). Thus, we hypothesize that agouti protein acts within the preputial gland to antagonize α-MSH-dependent signaling and thereby moderate the secretion of pheromone-like molecules into the urine that can influence the propensity for aggression in the host animal. Our finding of significantly increased preputial gland size in non-agouti mice, a feature known to depend on α-MSH signaling via local Mcr5 receptors (van der Kraan et al., 1998; Morgan and Cone, 2006) strongly supports an antagonistic function of agouti on Mcr5 signaling in this structure. Moreover, our findings that preputial glands of non-agouti mice contain significantly higher levels of at least one putative pheromone, 1-hexadecylacetate, suggest that agouti may act locally in the gland to alter the release of biologically active volatiles. The observation that the social behavior phenotype of non-agouti, but not agouti mice depends on the presence of an intact preputial gland argues for a role of such factors in the phenotype. One of the volatiles found to be increased in non-agouti preputial glands, 1-hexadecylacetate (Figure 3A), is found at higher levels in males than females (Zhang et al., 2007) and promotes social investigation in females when added to the urine of a castrated male a physiological levels (Zhang et al., 2008) suggesting that it is a mouse pheromone. However, at present it is not known which, if any, of these compounds are responsible for the behavioral phenotype we observed.

It may seem surprising that animals have developed a mechanism to regulate their own behavior via the secretion of compounds from their body, but one can speculate that such auto-pheromones might be particularly amenable to modulation by their mixing with pheromones and kairomones deriving from nearby individuals in such a way as to more appropriately adapt behavior coping strategies. Thus, we predict that the effect of agouti on behavior will be strongly modulated by the social context. We found no evidence of indirect genetic effects of agouti on social behavior (Figures S2, S4) suggesting that the effect of agouti is strictly autologous. However, we cannot rule out that under other circumstances agouti-dependent release of pheromones might alter the behavior of conspecifics. Our findings provide a genetic window on these mechanisms and open the door for their molecular understanding.

### Conflict of interest statement

The authors declare that the research was conducted in the absence of any commercial or financial relationships that could be construed as a potential conflict of interest.
