# Supplementary material for: Modulation of social behavior by the agouti pigmentation gene
Source: Front Behav Neurosci. 2014 Aug 1;8:259. doi: 10.3389/fnbeh.2014.00259 (PMC4117936; doi:10.3389/fnbeh.2014.00259)
Supplement: Supplementary file 1 [file Presentation1.PDF]

Supplementary Material

Modulation of social behavior by the agouti pigmentation gene

Valeria Carola[1,2], Emerald Perlas[2], Francesca Zonfrillo[2], Helena A. Soini[3], Milos V. Novotny[3], Cornelius T. Gross[2]*

[1] IRCCS Fondazione Santa Lucia, Via Fosso del Fiorano 63-64, 00143 Rome, Italy

[2] European Molecular Biology Laboratory (EMBL), Mouse Biology Unit, Via Ramarini 32, 00015 Monterotondo, Italy

[3] Institute for Pheromone Research and Department of Chemistry, Indiana University, 800 E. Kirkwood Ave., Bloomington, Indiana 47405, USA

*Correspondence: Cornelius T. Gross, European Molecular Biology Laboratory (EMBL), Mouse Biology Unit, Via Ramarini 32, 00015 Monterotondo, Italy.

Email: gross@embl.it

1. Supplementary Data

1.1 . Resident-intruder test with CD1 outbred mouse strain.

Male CD1 mice (12 weeks of age) were obtained from Charles River (Calco, Milan, Italy). Experimental conditions used to test the aggression-like behavior of CD1 mice were similar to the ones described in our paper (**Figure 1A-B**). Briefly agouti and non-agouti male mice were single housed before being exposed to resident-intruder test. The day of the test grouped-housed CD1 male mice were placed into a resident cage (non-agouti/agouti male) and the behavior of both mice observed for 40 minutes. The test was repeated only once and latency to the first

attack and number of attacks of the CD1 mouse were collected in real-time during the test (**Figure S3**).

2. Supplementary Figures and Tables

2.1 Supplementary Table 1. Levels of VOCs measured in preputial glands from non-agouti and agouti mice exposed to olfactory approach test (mean ± st. err; N = 11; *P < 0.05, **P < 0.01).

Preputial Gland VOCs	Non-agouti	Agouti	Correlation with behavior
Carvomenthene	*3.2 ± 0.57*	*1.77 ± 0.21**	
Limonene	8.45 ± 2.60	6.10 ± 0.54	
Decanoic acid	1.43 ± 0.24	1.62 ± 0.30	
Geranylacetone	0.76 ± 0.11	0.53 ± 0.11	
β-Farnesene	54.8 ± 18.7	66.1 ± 14.1	
a Sesquiterpene	0.50 ± 0.12	0.75 ± 0.15	
α-Farnesene	10.2 ± 3.80	12.8 ± 2.76	
Dodecanoic acid	5.09 ±1.02	8.44 ± 1.60	
Tetradecanoic acid	3.74 ± 0.96	6.42 ± 0.80	
Hexadecanoic acid	35.6 ± 6.27	51.7 ± 7.82	
1-Hexadecylacetate	*152.1 ± 22.2*	*86.6 ± 11.6**	
1-Heptadecylacetate	*4.37 ± 0.77*	*1.38 ± 0.19***	
Indane	*4.53 ± 0.99*	*9.12 ± 0.99**	X
A methylindan-1	*8.03 ± 1.70*	*14.8 ± 1.54**	X
A methylindan-2	5.91 ± 1.24	10.1 ± 1.92	X
1-Heptadecene	2.11 ± 0.33	1.34 ± 0.28	

2.2. Supplementary Figures

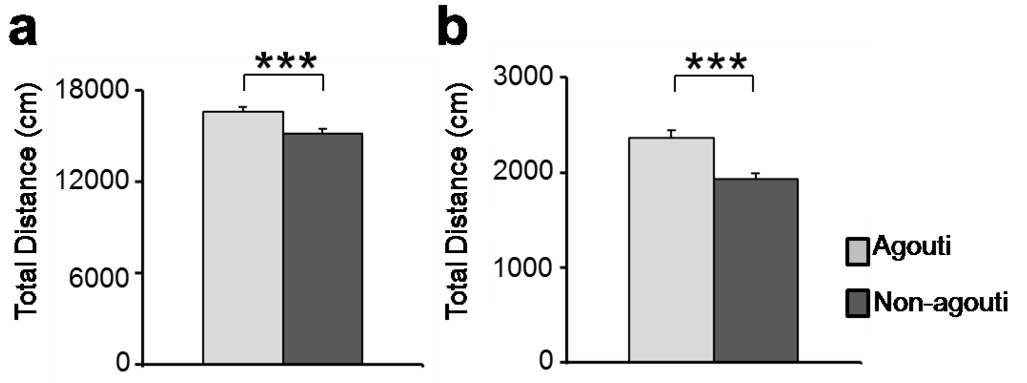

Supplementary Figure 1. Decreased locomotion in non-agouti mice. Non-agouti mice (N = 35) traveled significantly more than agouti littermates (N = 32) in the (**A**) open field and (**B**) elevated plus maze test (***P < 0.001).

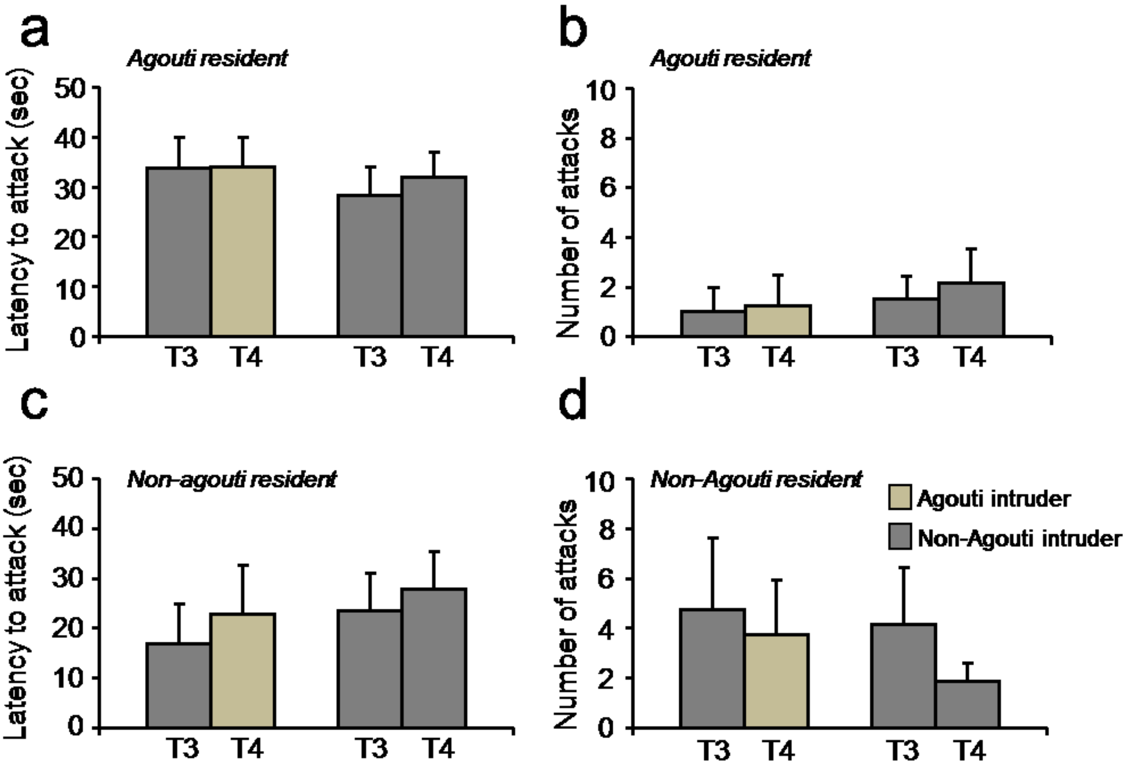

Supplementary Figure 2. Non-agouti mice attack more than agouti mice regardless of the genotype of the intruder. (**A-D**) In the fourth trial (T4) of the resident-intruder test (described in Figure 1A-B) there was not difference between the aggression-like behavior (latency to attack and number of attacks) observed in resident mice exposed to non-agouti intruder and resident mice exposed instead to agouti intruder. The aggression-like behavior observed in third trial (T3) of the resident-intruder test was similar to the behavior observed in the T4, suggesting the absence of a "within" effect in each group (non-agouti vs agouti resident mice).

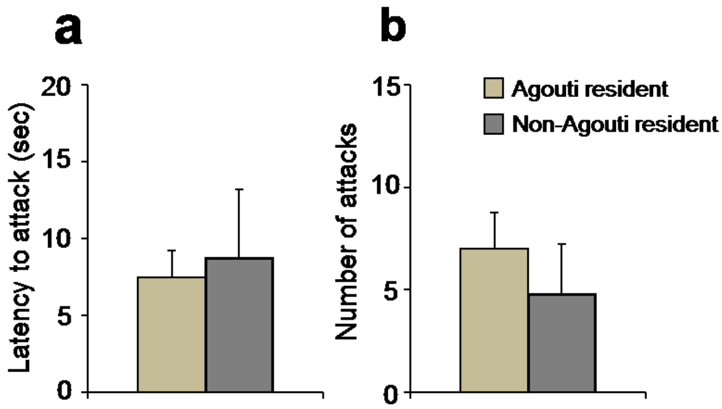

Supplementary Figure 3. Aggressive CD1 outbred mice similarly attack non-agouti and agouti mice. (A-B) CD1 mice showed similar aggression-like behavior (latency to attack and number of attacks) toward non-agouti and agouti mice (N = 15).

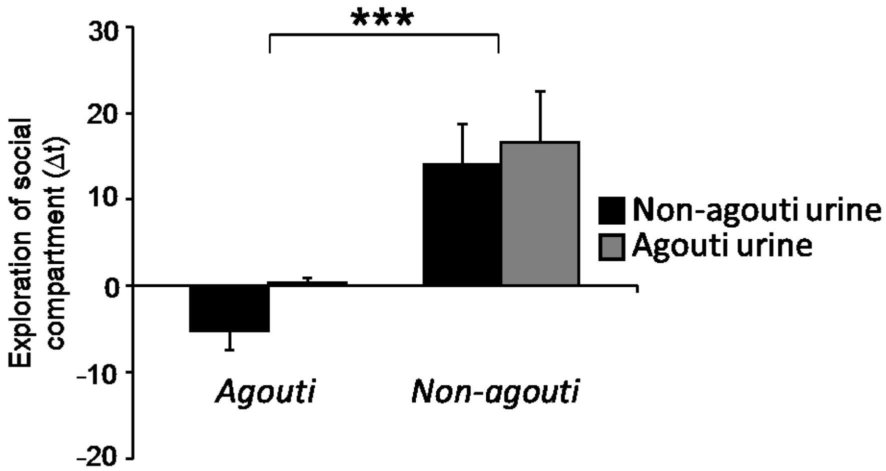

Supplementary Figure 4. Non-agouti mice showed a significantly greater preference for the urine-containing compartment than agouti littermates regardless of the type of urine used. (N = 35; ***P < 0.001).
